# Supplementary material for: Influence of Mycorrhiza on C:N:P Stoichiometry in Senesced Leaves
Source: J Fungi (Basel). 2023 May 18;9(5):588. doi: 10.3390/jof9050588 (PMC10220986; doi:10.3390/jof9050588)
Supplement: Supplementary file 1 [file jof-09-00588-s001.zip › Supplement table S1.pdf]

Table S1. The number of species in all groups in the database.

Groups	AM	AM+ECM	ECM	Total
**Total**	244	45	108	397
**Boreal**	48	14	32	94
**Temperate**	81	27	80	188
**Tropical**	103	14	16	133
**Woody plant**	175	44	108	327
**Deciduous**	91	23	49	163
**Evergreen**	89	21	59	169
**Broadleaf**	161	39	71	271
**Conifer**	13	5	37	55
